# Diagnosis of *Salmonella enterica*-induced septic arthritis in a healthy child using metagenomic next-generation sequencing: a case report

**DOI:** 10.3389/fped.2025.1704234

**Published:** 2025-11-19

**Authors:** Min Ren, Qiong Liao

**Affiliations:** Department of Pediatric Infectious Diseases, West China Second University Hospital, Key Laboratory of Birth Defects and Related Diseases of Women and Children (Sichuan University), Ministry of Education, Chengdu, China

**Keywords:** mNGS, *salmonella enterica*, septic arthritis, healthy child, case report

## Abstract

*Salmonella enterica*-induced arthritis frequently manifests in children with malignancies, sickle cell disease, immunodeficiency, or undergoing immunosuppressive therapy; however, its incidence in healthy children is rare. Here, we present a case of septic arthritis resulting from *S. enterica* infection in a previously healthy child, diagnosed utilizing metagenomic next-generation sequencing (mNGS). This case underscores the utility of mNGS in the clinical identification of *S. enterica* arthritis. Particularly in scenarios where the causative pathogen remains unidentified, mNGS emerges as a pivotal adjunctive diagnostic modality for uncommon pathogens.

## Introduction

*Salmonella*, a Gram-negative bacillus within the *Enterobacteriaceae* family, is a significant global pathogen responsible for foodborne illnesses mainly transmitted through the fecal-oral route (1). Infections typically manifest as gastroenteritis, bacteremia, or localized infections. Although relatively uncommon, *Salmonella enterica*-induced arthritis presents clinically with a fever incidence of 0.1%–0.2% in pediatric septic arthritis, predominantly affecting the hip joint (2). This condition often occurs in children with underlying conditions such as sickle cell disease, immunodeficiency, or those undergoing immunosuppressive therapy (3, 4). Septic arthritis stemming from *S. enterica* infection is rare in healthy children. The advent of metagenomic next-generation sequencing (mNGS) presents a novel approach to pathogen detection. This article presents a case study of septic arthritis caused by *S. enterica* infection diagnosed via mNGS in an otherwise healthy child.

## Case presentation

A 3-year-old boy was admitted with swelling and pain in his right knee joint persisting for 13 days, accompanied by a 3-day fever. Prior to symptom onset, he accidentally scratched the skin over his right knee while playing. The injury resulted in local skin abrasions and bleeding but did not impede his ability to walk. The following day, he experienced pain and restricted mobility in the right knee, along with a limp. Initially, there were no signs of fever, chills, redness, or swelling in the affected joint. He received initial treatment at a local orthopedic hospital, where plain radiographs revealed no abnormalities, and a diagnosis of synovitis was considered. Despite over 10 h of topical traditional Chinese medicine application, there was no improvement in pain or mobility. Two days after the onset of symptoms, the patient exhibited significant redness, swelling, warmth, and pain in the right knee joint, accompanied by worsened mobility and the onset of fever, peaking at 39.5 °C. Magnetic resonance imaging (MRI) revealed a small effusion within the knee joint cavity, confirming synovitis. Intravenous Cefathiamidine was administered for anti-infective therapy. After 48 h, the patient's temperature normalized, and swelling decreased, though pain and limited mobility persisted. The local skin temperature of the right knee remained elevated compared to the healthy side, with symptoms gradually improving over a 10-day treatment period.

**Figure 1:**
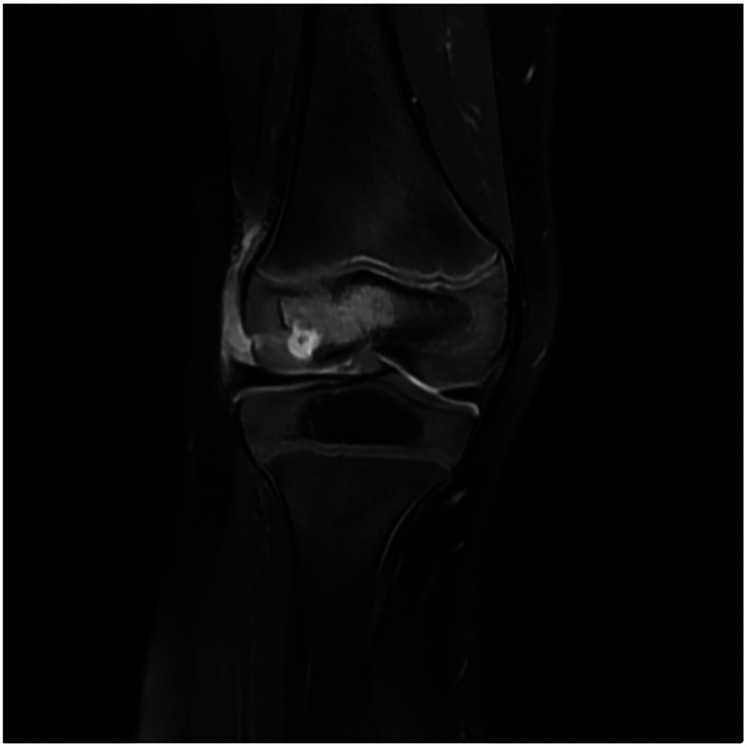
The image demonstrates abnormal hyperintense signals with rim enhancement in the epiphysis of the left distal femur, consistent with septic arthritis-related bone marrow edema and inflammatory changes.

**Figure 2:**
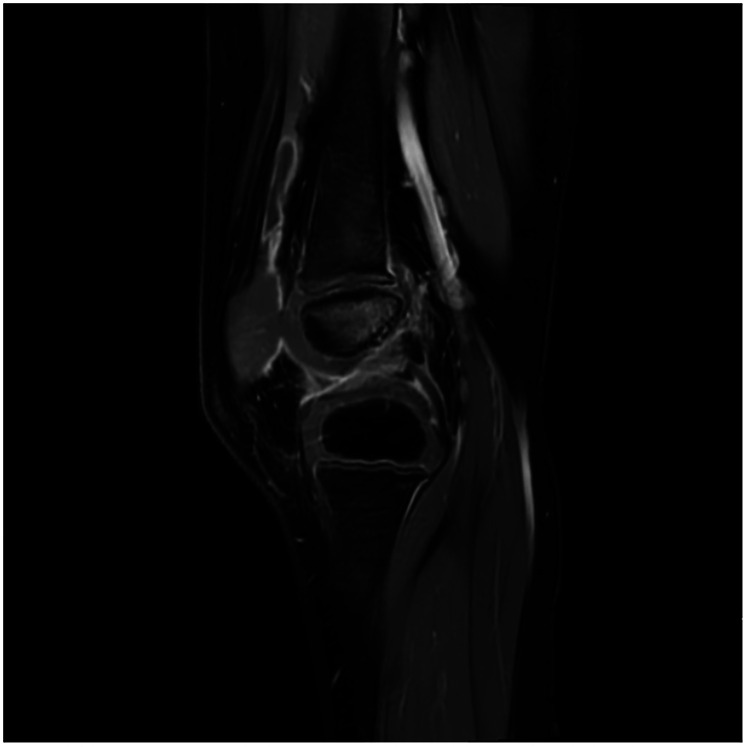
The image reveals prominent joint effusion, synovial enhancement, and surrounding soft tissue edema, consistent with septic arthritis-related inflammatory changes.

**Figure 3:**
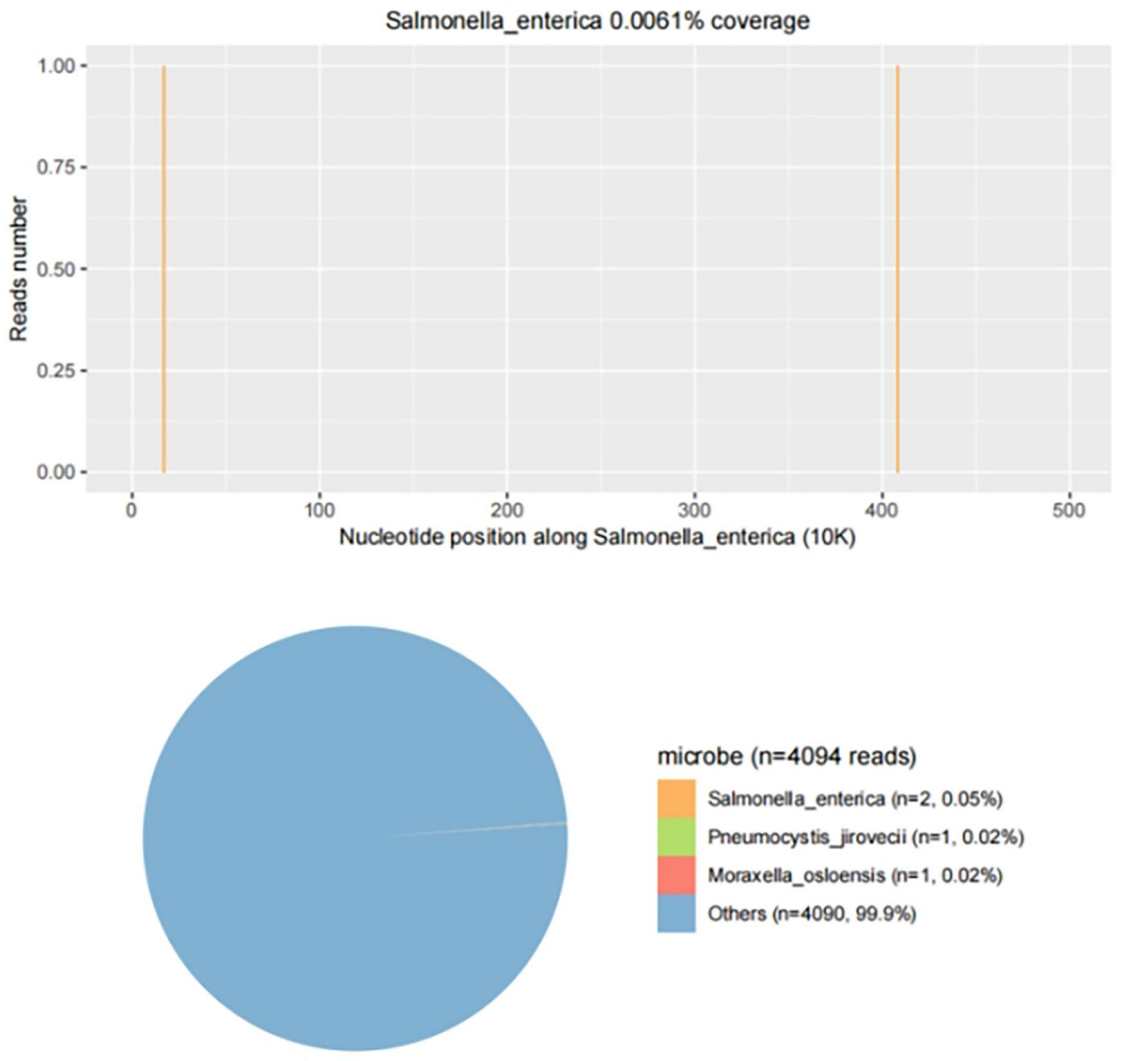
mNGS results from joint fluid. Genome coverage and the number of specific reads detected for *Salmonella enterica* are shown. Two unique reads mapped to the *S. enterica* genome out of 4,094 microbial reads, with a genome coverage of 0.0061%.

**Figure 4:**
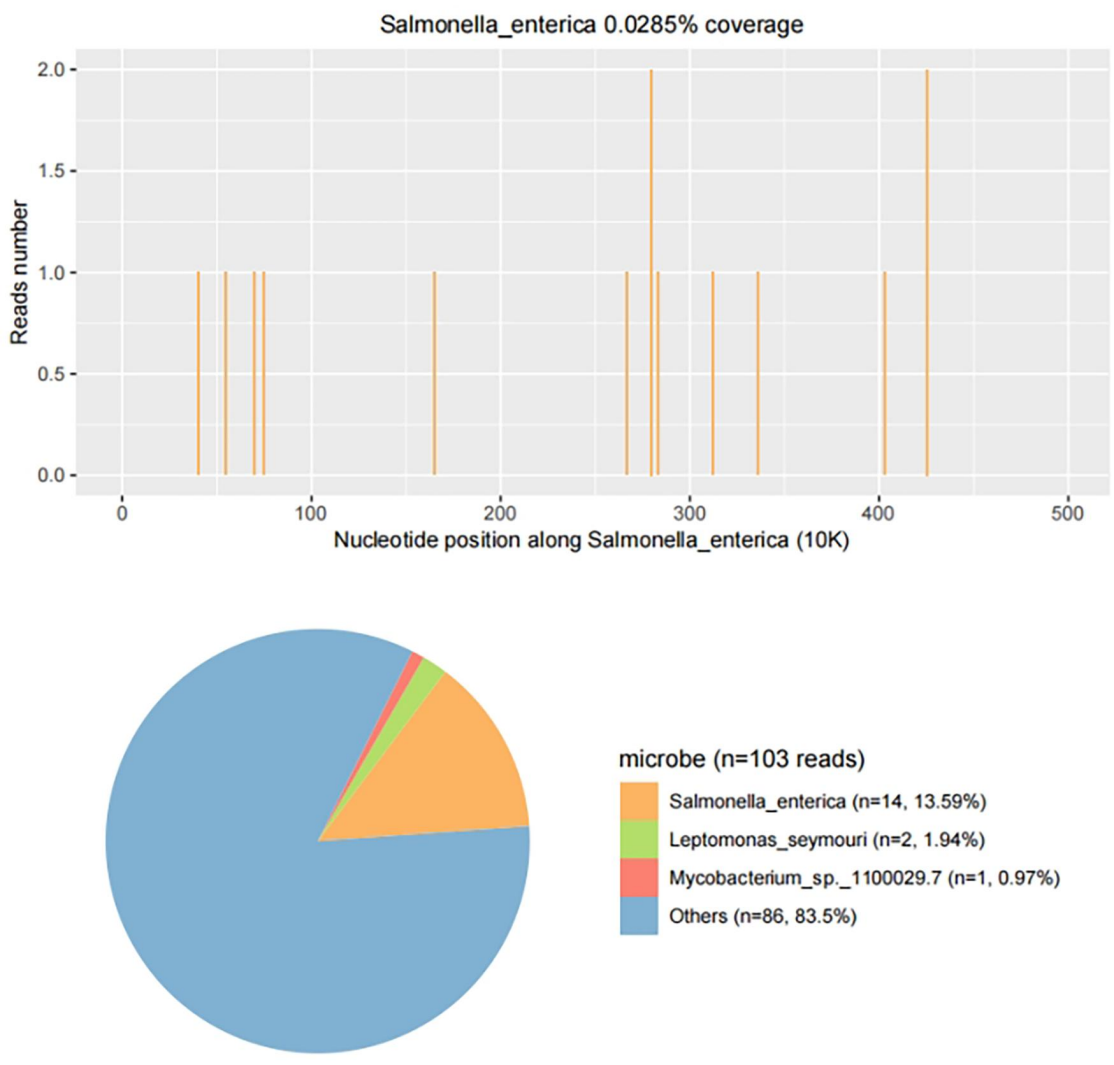
mNGS results from joint fluid. Genome coverage and the number of specific reads detected for *Salmonella enterica* are shown. A total of 14 reads mapped to the *S. enterica* genome out of 103 microbial reads, with a genome coverage of 0.0285%.

Upon admission, the child presented with vital signs within normal ranges: Temperature, 36.3 °C; heart rate, 97 beats/min; respiratory rate, 19 breaths/min; and blood pressure, 86/53 mmHg. The right knee showed limited extension and was swollen with increased skin temperature and tenderness. The patellar taping test was inconclusive. Laboratory findings indicated: white blood cell count 12.0 × 10^9^/L,hemoglobin,92 g/L, blood platelet count 656 × 10^9^/L, C-reactive protein 26.2 mg/L,and erythrocyte sedimentation rate: 107 mm/h. Liver and kidney function, autoantibodies, Interferon Gamma Release Assay, tuberculin skin test, and blood cultures were negative. Empiric antibacterial therapy with Clindamycin was administered intravenously for 4 days, resulting in recurrent fever for the initial 2 days, peaking at 38.3 °C. Fever subsided after 2 days, but joint symptoms persisted. Due to suspected methicillin-sensitive *Staphylococcus aureus* (MSSA) infection-induced purulent arthritis, treatment shifted to intravenous oxacillin for 5 days. However, fever recurred after 4 days, prompting consideration of septic arthritis from methicillin-resistant *Staphylococcus aureus* (MRSA) or other gram-negative bacteria. Antibiotics were escalated to intravenous oxacillin for 5 days. Therefore, the antibiotic regimen was upgraded to intravenous vancomycin and meropenem. Despite this, the child continued to experience a recurrent high fever. Enhanced MRI of the right knee joint revealed abnormal signals in the distal femoral epiphysis involving the adjacent epiphyseal cartilage with local penetration of the epiphyseal cartilage into the joint cavity, lateral collateral ligament, and anterior cruciate ligament. Multiple areas of synovial thickening and enhancement were observed, with slight swelling of the soft tissue around the right knee joint and effusion in the suprapatellar bursa and joint cavity, suggesting infectious lesions (Figures 1, 2). Chest, abdominal, and cardiac ultrasonography revealed no evidence of migratory lesions or thrombosis in the limbs. As blood cultures were negative, blood was sent for mNGS to confirm the pathogen, which detected *Salmonella enterica* (Figure 3). Similarly, mNGS testing of joint cavity effusion aspirates yielded *Salmonella enterica* (Figure 4), whereas cultures of the joint cavity effusion were negative. Metagenomic next-generation sequencing (mNGS) analysis showed that *Salmonella enterica* was detected in both blood and joint fluid samples. The genome coverage of *Salmonella enterica* was 0.0061% (2/4,094) in blood and 0.0285% (14/103) in joint fluid, with 2 and 14 microbial reads identified respectively (The mNGS workflow and bioinformatics description are provided in the Supplementary Materials). Following the mNGS results, the treatment was adjusted to ceftriaxone as an anti-infective therapy,considering its excellent tissue penetration. After 48 h of ceftriaxone treatment, the child's fever improved, and the swelling and pain in the right knee joint were gradually alleviated. Four days later, the patient's body temperature normalized. The child received intravenous ceftriaxone for 4 weeks and remained stable during follow-up visits. The CRP and ESR returned to normal, and the child was discharged on oral cefpodoxime during outpatient follow-up. The child's condition stabilized and recovered with a normal walking gait. Whole-genome sequencing revealed negative results for the child and his parents during the follow-up visit after six months, with no recurrence of symptoms.The detailed diagnosis and treatment process are shown in Table 1.

**Table 1:** Diagnosis and treatment process.

Standardized time point	Key events (symptoms/examinations)	Key diagnostic findings	Key treatments
Day 0 (Pre-injury)	Scratched right knee skin during play	None	No specific treatment
Day 1 (the day after injury)	Right knee pain, limited mobility, limping; underwent orthopedic x-ray examination	Orthopedic x-ray showed no abnormality	Topical Chinese medicine (used for >10 h, ineffective)
Day 2 (Symptom aggravation)	Redness, swelling, increased skin temperature, pain in the right knee, accompanied by fever (maximum body temperature 39.5 °C); underwent knee MRI examination	Knee MRI indicated joint effusion	Intravenous cefathiamidine (fever resolved in 48 h, continued for 10 days)
Hospital Day 1	Limited right knee extension, swelling, tenderness; underwent inflammatory marker detection and blood culture examination	Elevated inflammatory markers; Negative blood culture	No treatment adjustment
Hospital Days 1–4	nitial recurrent fever (maximum body temperature 38.3 °C); joint symptoms persisted after fever subsided	No new diagnosis	Intravenous clindamycin (poor efficacy)
Hospital Days 5–9	Recurrent fever	Suspected suppurative arthritis caused by methicillin-sensitive Staphylococcus aureus (MSSA) infection	Intravenous oxacillin (5 d total, fever recurred on Day 4)
Hospital Days 10–14	Persistent high fever; underwent enhanced knee MRI examination	Enhanced knee MRI indicated infectious lesions	Intravenous vancomycin + meropenem (ineffective)
Hospital Day 15 (approx.)	Underwent metagenomic next-generation sequencing (mNGS) of blood and joint fluid	*Salmonella enterica* detected by blood and joint fluid mNGS	Intravenous ceftriaxone (fever resolved in 48 h)
Ceftriaxone treatment period (4 weeks)	Body temperature returned to normal on the 4th day of treatment; inflammatory markers rechecked during follow-up	Inflammatory markers gradually returned to normal during follow-up	Intravenous ceftriaxone for 4 weeks; oral cefpodoxime after discharge
6 months after discharge	Normal walking gait, no symptom recurrence; the child and parents underwent whole-genome sequencing	Whole-genome sequencing results of the child and parents were negative	Follow-up completed, no further treatment required

## Discussion

*Salmonella,* a zoonotic intestinal pathogen, is primarily categorized into Salmonella typhi, Salmonella paratyphi, and non-typhoidal Salmonella (NTS) (5). The former two primarily cause typhoid fever, while the latter mainly induces non-specific enteritis and invasive extraintestinal infections. *Salmonella enterica* falls under the NTS bacteria family. Children typically contract NTS infection through ingestion or contact with contaminated substances, presenting symptoms such as diarrhea (6). NTS infections can disseminate via the bloodstream, leading to localized infections, with septic arthritis being a rare complication (7). Most cases of Salmonella arthritis affect single joints, while others arise post-trauma or after prosthetic joint replacement (8, 9). Notably, the absence of a prior unclean diet, diarrheal symptoms, and similar manifestations in cohabiting family members is a unique clinical feature of our case. This differs from the typical clinical profile of Salmonella arthritis—where gastrointestinal symptoms or epidemiological links (e.g., family exposure) are commonly reported (5, 10), further expanding the phenotypic spectrum of this rare infection. Initial trauma management involved topical traditional Chinese medicine, but the possibility of infection through the wound cannot be dismissed.

This case underscores the clinical challenge of diagnosing culture-negative Salmonella arthritis in children. As timely diagnosis of pediatric septic arthritis is critical to prevent permanent disability, reliance solely on traditional culture methods may lead to delays—especially given the low blood culture positive rate (10%–15%) for Salmonella arthritis (11). Although microbial culture remains the gold standard for pathogen identification, mNGS offers distinct advantages: shorter turnaround time and higher sensitivity (61.9% vs. 45.2% in a 42-patient prospective study) (12, 13). Consistent with previous suggestions that genetic sequencing is optimal for suspected infections with negative etiological tests (14). A notion supported by our case, where mNGS confirmed *Salmonella enterica* infection despite negative blood, bone marrow, and joint fluid cultures. Importantly, to our knowledge, this is the first case report documenting the diagnosis of Salmonella arthritis using mNGS, expanding the utility of this technology for rare, culture-negative pediatric osteoarticular infections.

Third-generation cephalosporins are the preferred initial antibiotics for treating NTS infection in children. Quinolones are generally not recommended for pediatric use, though they might be considered in severe infections where other antibiotics are unavailable (15). Carbapenems are effective against multidrug-resistant Salmonella infections. However, meropenem proved ineffective in this case, possibly due to inadequate local concentration in the joints. Initial treatment with clindamycin and cefotiam did not significantly alleviate joint swelling and pain, likely due to poor bacterial sensitivity. Ceftriaxone was chosen for its superior tissue penetration and efficacy.

Septic arthritis caused by NTS generally carries a more favorable prognosis compared to that caused by other Gram-negative bacteria. When empirical anti-infective treatment proves ineffective, consideration should be given to the possibility of Salmonella arthritis, highlighting the necessity of pathogen detection. Salmonella infections are typically identified through fluid or stool cultures; however, in cases where conventional microbial cultures yield negative results, mNGS can be employed to confirm the presence of the pathogen. Although susceptibility test results may not be readily available, third-generation cephalosporins are currently favored as treatment options. In similar scenarios, ceftriaxone, in alignment with local epidemiology and bacterial resistance rates, may be contemplated to avert delays in achieving effective treatment in future. Ensuring adequate and complete therapy courses is paramount for effectively treating lesions and preventing recurrence.

## Conclusion

Septic arthritis caused by Salmonella enteritidis is extremely rare in healthy children, with atypical clinical manifestations. Its diagnosis is challenging when conventional culture results are negative. For culture-negative septic arthritis in children, metagenomic next-generation sequencing (mNGS) should be regarded as an important auxiliary diagnostic tool. Ceftriaxone may be an effective therapeutic option for Salmonella enteritidis-associated arthritis in children. When empirical treatment fails and culture results are negative, clinicians should consider utilizing mNGS even for healthy children without typical epidemiological clues.

## Data Availability

The original contributions presented in the study are included in the article/Supplementary Material, further inquiries can be directed to the corresponding author.
