# Supplementary material for: Diagnosis of Salmonella enterica-induced septic arthritis in a healthy child using metagenomic next-generation sequencing: a case report
Source: Front Pediatr. 2025 Nov 19;13:1704234. doi: 10.3389/fped.2025.1704234 (PMC12672467; doi:10.3389/fped.2025.1704234)
Supplement: Supplementary file 1 [file Datasheet1.pdf]

1. Nucleic Acid Extraction

Genomic DNA from each sample was extracted using the Nucleic Acid Extraction Reagent Kit (Cat. No. YGZZ014, HugoBiotech, Beijing, China) following the manufacturer's standard protocol. Extraction was performed in a sterile biosafety cabinet to minimize cross-contamination, and all reagents were pre-checked for sterility.

2. Library Construction

Sequencing libraries were constructed using the Universal Sequencing Reaction Kit (Cat. No. YGZZ005, HugoBiotech, Beijing, China). Briefly, extracted DNA was fragmented, and sequencing adapters were ligated according to the kit instructions. Library concentration was quantified using a Qubit 4.0 Fluorometer (Thermo Fisher Scientific, Waltham, USA) with the dsDNA HS Assay Kit. Library integrity and average fragment size were assessed using an Agilent 2100 Bioanalyzer (Agilent Technologies, Santa Clara, USA); only libraries with an RNA Integrity Number (RIN)-equivalent score ≥ 7.0 were used for sequencing.

3. Sequencing

After passing quality control, libraries were sequenced on the Illumina 550DX platform (Illumina, Inc., San Diego, USA) using single-end 75 bp read mode. Each sequencing batch included:

Negative controls (NTC): Sterile deionized water, processed through the entire extraction and library construction workflow to monitor environmental contamination.

Positive controls: Synthetic microbial DNA fragments of known quantity, used to validate the sensitivity and accuracy of the detection pipeline.

4. Bioinformatics Analysis Pipeline

Raw sequencing data were processed using the following steps, with software versions and parameters specified for reproducibility:

4.1 Raw Data Conversion and Demultiplexing

Raw data in "bcl" format were converted to FASTQ format and demultiplexed (to split data for individual samples in a batch) using bcl2fastq software (v2.20.0.422;

Illumina, Inc.) with default parameters.

4.2 Quality Control of Sequences

High-quality clean reads were generated using fastp (v0.24.0) [1] with default parameters, which included:

Trimming of adapter sequences.

Removal of low-quality reads (Phred quality score < 20 over 50% of bases).

Exclusion of sequences containing a high percentage of ambiguous “N” bases.

Only reads with a minimum length of 50 bp were retained for downstream analysis.

4.3 Host Sequence Removal

Clean reads were aligned to the human reference genome (GRCh38.101) using BWA (v0.7.15) [2] in “mem” mode to filter out host-derived sequences. Reads that mapped to the human genome (alignment score ≥ 30) were discarded; unmapped reads were retained for microbial identification.

4.4 Microbial Identification

Retained (non-host) reads were aligned to an in-house microbial genome database using BWA (v0.7.15). This database was compiled from publicly available sequences in NCBI RefSeq and GenBank, covering bacteria, viruses, fungi, and parasites. Microbial species were identified based on alignment results, and two key metrics were calculated for each detected microorganism:

Number of unique aligned reads (reads mapping exclusively to that microorganism).

RPM (reads per million clean reads), defined as (number of unique reads / total clean reads per sample) × 10^6.

5. Criteria for Positive mNGS Results

A microorganism was considered “positive” based on the following criteria:

For bacteria (excluding *Mycobacterium*), fungi (excluding *Cryptococcus*), and parasites:(1) The genome coverage of unique reads mapped to the microorganism ranked in the top 10 among all microbes of the same category, and the microorganism was not detected in the NTC; or(2) The ratio of sample RPM to NTC RPM (RPM_{sample}/RPM_{ntc}) was > 10 (with $RPM_{ntc} \neq 0$).

For viruses, *Mycobacterium tuberculosis*, and *Cryptococcus*:(1) The microorganism

was not detected in the NTC, and at least 1 unique read mapped to the species-level genome; or(2) $RPM_{sample}/RPM_{nt}c > 5$ (with $RPM_{nt}c \neq 0$).

References

[1] Chen S, Zhou Y, Chen Y, Gu J. fastp: an ultra-fast all-in-one FASTQ preprocessor. *Bioinformatics*. 2018;34(17):i884–i890. doi:10.1093/bioinformatics/bty560.

[2] Li H, Durbin R. Fast and accurate long-read alignment with Burrows-Wheeler transform. *Bioinformatics*. 2010;26(5):589–595. doi:10.1093/bioinformatics/btp698.
